# Characterization of viral diversity in wild marmot blood from the Qinghai–Tibet Plateau

**DOI:** 10.3389/fmicb.2026.1668126

**Published:** 2026-02-06

**Authors:** Haisheng Wu, Xiaojie Jiang, Yuan Xi, Songyi Ning, Hailian Wu, Wenyuan Xin, Wenxuan Peng, Shengjun Wang, Wen Zhang

**Affiliations:** 1 Department of Microbiology, School of Medicine, Jiangsu University, Zhenjiang, Jiangsu, China; 2 Qinghai Institute for Endemic Disease Prevention and Control, Xining, China; 3 Department of Clinical Laboratory, Wuxi Blood Center, Wuxi, Jiangsu, China

**Keywords:** blood, Qinghai–Tibet Plateau, viral diversity, wild marmots, zoonotic potential

## Abstract

**Introduction:**

This study aimed to characterize the viral diversity in the blood of marmots in the Qinghai-Tibet Plateau region, to assess their role as potential viral reservoirs and evaluate the potential implications for wildlife and human health.

**Methodology:**

Seventy marmot blood samples were collected from Yushu and Guoluo Prefectures in Qinghai Province. Viral communities were comprehensively analyzed using high-throughput sequencing and bioinformatics techniques.

**Results:**

Analysis identified a wide range of viral families, including *Anelloviridae*, *Flaviviridae*, *Parvoviridae*, and *Polyomaviridae*, and revealed multiple novel viral sequences. Notably, we documented the first evidence of *Anelloviridae* in marmot serum; phylogenetic analysis indicated these sequences cluster with those from marmot feces and tissues, suggesting a natural host relationship. A critical finding was the detection of Tick-borne encephalitis virus, with a sequence highly similar to human-derived strains, implying potential involvement of marmots in the transmission cycle. Furthermore, identification of a novel polyomavirus was supported by prediction of all main large tumor antigen functional domains and motifs, including a putative nuclear localization signal between its LXCXE motif and origin-binding domain, typical of mammalian-infecting polyomaviruses. Comparative analysis revealed significant regional differences in viral diversity between sampling areas, potentially linked to local ecological factors.

**Discussion:**

This study significantly expands the known viral diversity in marmots and underscores their role as potential zoonotic reservoirs. However, the functional and pathogenic implications of these viruses require further experimental validation. These findings highlight the importance of ongoing wildlife surveillance for understanding viral ecology and mitigating emerging public health risks.

## 1 Introduction

Marmots (genus *Marmota*) are large ground squirrels that are found primarily in temperate regions of North America, Europe and Asia (Arnold, 2019). These rodents perform vital ecosystem roles by improving soil aeration while serving as key prey for top predators including snow leopards (*Panthera uncia*) and wolves (*Canis lupus*), thus sustaining trophic dynamics across Central Asia and China’s Qinghai–Tibet Plateau (Lyngdoh et al., 2014; Karimov et al., 2018). Particularly, the Himalayan marmot, as an important rodent species of the Tibetan Plateau and surrounding areas, is not only a major reservoir for bacterial diseases such as plague but also harbors a variety of viral pathogens, including bloodborne viruses such as hepatitis virus (Millman et al., 1984; Ben-Ari et al., 2012; Ma et al., 2024). These viruses may affect their health and pose risks to other wildlife and even humans. Specifically, marmots may serve as reservoirs for several zoonotic viruses, making the monitoring of their viral load and health status crucial for wildlife conservation and public health (Luis et al., 2013).

Marmots are exposed to several bloodborne viruses, which may be transmitted via vector organisms such as ticks, fleas, and other ectoparasites (Islam et al., 2021). Studying these viruses in marmots is important for understanding the potential risks they pose to both wildlife populations and human health, particularly in regions where marmots live in close proximity to human settlements (Xi et al., 2022). Research has shown that marmots can harbor several families of viruses, including flaviviruses (Mlera and Bloom, 2018), Hepatitis B (Liu et al., 2020; Michalak, 2020), and even coronaviruses (Moreno et al., 2024). Understanding these viruses in marmots is key to preventing potential outbreaks and mitigating health risks.

Tick-borne encephalitis virus (TBEV) is a flavivirus transmitted by ticks and has been found in various wild rodents, including marmots (Lindquist and Vapalahti, 2008). The detection of the Himalayan subtype of Tick-borne encephalitis virus in marmots suggests that they may serve as amplifying hosts in the arbovirus transmission chain (Dai et al., 2018). This virus is transmitted through tick bites and is highly pathogenic to the human central nervous system (Bogovic and Strle, 2015). The detection of TBEV in marmots highlights their potential role in maintaining the virus in natural foci. Moreover, the presence of the Himalayan subtype of TBEV in marmots suggests that these animals may act as a bridge for the virus to spread across different regions. This is particularly concerning given the virus’s high pathogenicity in humans, causing severe neurological disorders such as encephalitis and meningitis. Understanding the role of marmots in the blood-mediated transmission of TBEV is crucial for developing effective surveillance and control strategies to mitigate the risk of human infection.

Detecting blood-borne viruses in marmots typically requires a combination of molecular and serological methods. Commonly used detection techniques include serological assays (Svoboda et al., 2014). Additionally, next-generation sequencing (NGS) methods enable comprehensive high-throughput analysis of viral diversity in marmot blood samples, serving as an effective tool for discovering novel viruses or detecting low-abundance viruses (Zhu et al., 2020; Hilt and Ferrieri, 2022). These comprehensive sampling and processing methods aim to enhance our understanding of viral dynamics in wildlife, reduce the risk of viral spillover, and provide critical support for public health and ecological conservation efforts.

Understanding the viral ecology of marmots is crucial for better risk assessment, particularly in regions where marmot habitats overlap with human activities (He et al., 2021). Monitoring blood-borne viruses in marmots helps track viral transmission dynamics, especially in areas where marmots serve as reservoir hosts for tick-borne viruses such as TBEV (Kwasnik et al., 2023; Pustijanac et al., 2023). To address these knowledge gaps, we conducted an analysis of marmot blood samples from Yushu Prefecture and Guoluo Prefecture in Qinghai Province. This study aims to enhance our understanding of viral dynamics in wildlife, reduce the risk of viral spillover, and provide support for public health and ecological conservation efforts.

## 2 Methods

### 2.1 Sample collection

We collected blood samples from 50 marmots in Yushu Prefecture and 20 marmots in Guoluo Prefecture, Qinghai Province. These marmots (*Marmota himalayana*) were captured in their natural habitats using humane live traps. In accordance with ethical guidelines, all marmots were anesthetized, and blood samples were obtained via cardiac puncture. After centrifugation, the supernatant was discarded, and the samples were stored at −80 °C for further analysis. Marmots in this study were captured using ketamine hydrochloride as the anesthetic and were subsequently euthanized. All animal procedures in this study were conducted in accordance with the guidelines of the Ethics Committee of Jiangsu University and were approved (Approval No. JSDX20231007002).

All samples were resuspended in Dulbecco’s phosphate buffered saline (DPBS), homogenized, and subjected to three freeze–thaw cycles. The supernatants were collected after centrifugation at 15,000 × g for 10 min at 4 °C.

### 2.2 Library construction

Each supernatant (500 μL) was filtered through a 0.45-μm syringe filter (Millipore) to remove large cell-sized particles and subsequently centrifuged at 12,000 × g for 5 min to collect the filtrate enriched in viral particles. To degrade unprotected nucleic acids, the filtrate was incubated with a dual-enzyme cocktail of DNase I and RNase A at 37 °C for 60 min (Zhang et al., 2014, 2016; Zhao et al., 2022). Detailed reagent specifications are provided in Table 1. Total nucleic acids (RNA and DNA) protected within viral capsids were then extracted using either the FastPure Viral DNA/RNA Mini Kit (Vazyme) or the QiAamp Viral RNA Mini Kit, following the manufacturers’ protocols. Reverse transcription was performed on the extracted nucleic acids using SuperScript III or SuperScript IV reverse transcriptase (Invitrogen) with random hexamer primers. For single-stranded DNA (ssDNA) viruses, ssDNA was converted to double-stranded DNA (dsDNA) using the Klenow fragment polymerase (New England BioLabs).

**Table 1:** Nuclease digestion system.

Reagents	Volume
10× buffer cocktail	20.0 μL
Enzyme cocktail I	7.0 μL
Enzyme cocktail II	3.0 μL
Benzonase (250 U/μL)	3.0 μL
RNase A	0.5 μL
Liquid sample	166.5 μL
Total	200.0 μL

The resulting nucleic acids were prepared for sequencing using the Illumina Nextera XT DNA Sample Preparation Kit, with dual barcoding applied to distinguish between samples. A total of 70 pools were constructed, depending on the study design, and sequenced on the Illumina NovaSeq 6000 platform, generating 250-bp paired-end reads (Liu et al., 2016).

### 2.3 Bioinformatics analysis

The 250-bp paired-end reads generated for each pool were debarcoded using Illumina’s vendor software. An in-house analysis pipeline running on a 32-node Linux cluster was used to process the data. Reads were considered duplicates if bases 5 to 55 were identical and only one random copy of duplicates was kept. Clonal reads were removed and low sequencing quality tails were trimmed using Phred quality score 30 as the threshold. Adaptors were trimmed using the default parameters of VecScreen which is NCBI BLASTn with specialized parameters designed for adapter removal. The cleaned reads were then subjected to a DIAMOND BLASTx search against an in-house non-virus non-redundant (NVNR) protein database (Buchfink et al., 2015), which was compiled from NCBI nr fasta files by excluding viral taxonomic annotations, to eliminate false positive viral hits. Taxonomic classification of the DIAMOND results was parsed using MEGAN, with the lowest common ancestor (LCA) assignment performed under default settings. To further identify viral sequences, the cleaned reads were *de novo* assembled using Geneious Prime v2019.0 (Biomatters Ltd.) with default parameters. The resulting contigs and singlet sequences were compared against a viral proteome database using BLASTx with an *E*-value threshold of <10^−5^ to classify virus types and filter out non-viral sequences. The viral proteome database was constructed using the NCBI virus reference proteome (available at http://ftp.ncbi.nih.gov/refseq/release/viral/) and viral protein sequences extracted from the NCBI nr FASTA files, categorized based on annotation taxonomy within the Virus Kingdom (Skewes-Cox et al., 2014; Deng et al., 2015).

To identify more distant viral protein similarities, contigs without significant BLASTx hits were further analyzed against viral protein families in the vFam database using HMMER3 (Johnson et al., 2010; Finn et al., 2011). Open reading frames (ORFs) within the viral genomes were predicted by integrating the BLASTx search results with Geneious Prime software. Protein domains were annotated using the NCBI Conserved Domain Search tool with an *E*-value threshold of <10^−5^. This comprehensive approach ensured the accurate identification and characterization of viral sequences while minimizing false positives and enhancing the detection of remote viral homologs. Protein sequences were analyzed with cNLS Mapper^1^ to identify putative cNLSs (Kosugi et al., 2009). cNLS Mapper (last updated on 2012/11/7) was used with uniform threshold settings: monopartite NLS ≥3, bipartite NLS ≥3. Structural models were predicted using the AlphaFold3 Server and the ColabFold v1.5.5: AlphaFold2 using MMseqs2 (Mirdita et al., 2022; Abramson et al., 2024). Conduct domain and motif analysis using ProSite^2^ and ELM.^3^

### 2.4 Viral sequences acquisition

To generate high-quality viral genomes or genomic segments, *de novo* assembly and reference mapping were conducted in Geneious Prime v2019.0 using the assembled contigs and unassembled reads with known taxonomic assignments obtained from the previous analysis stage. Additionally, Geneious Prime v2019.0 was utilized for genome annotation and ORF prediction, enabling comprehensive characterization of the viral sequences.

### 2.5 Statistical analysis

Statistical analyses were performed using R v4.2.1 and MEGAN v6.22.2. All reads in the quality-controlled data with sequence lengths greater than 50 bp were aligned to the viral proteome database using BLASTx (method as described above). The BLASTx results were then imported into the MEGAN software to generate rarefaction curves, visualizing differences in viral community composition (Huson et al., 2007). To visualize the viral community structure and richness, the R packages *pheatmap* and *vegan* were employed. Differences in viral communities were graphically represented using the *ggplot2* package in R. Viral community alpha diversity was analyzed at the family level, with statistical significance assessed using the Kruskal–Wallis test, followed by pairwise Wilcoxon rank-sum tests for group comparisons. A *p*-value threshold of <0.05 was applied to determine statistical significance.

### 2.6 Phylogenetic analysis

Phylogenetic analyses were performed using the predicted protein sequences of the viruses identified in this study, along with approximately the top 50 protein sequences retrieved by BLASTx, as well as reference protein sequences from diverse host species or different viral genera obtained from the NCBI GenBank database (Shan et al., 2022). Related protein sequences were aligned using MUSCLE in MEGA version 10.1.8 with default settings (Kumar et al., 2018). Sites containing more than 50% gaps were temporarily removed from alignments. Bayesian inference trees were constructed using MrBayes version 3.2.7 (Ronquist et al., 2012), employing two concurrent runs of Markov chain Monte Carlo (MCMC) sampling. For protein-based phylogenetic analysis, the mixed amino acid model was specified using the command “prset aamodelpr = mixed.” The runs were terminated when the standard deviation of split frequencies fell below 0.01, and the first 25% of the trees were discarded as burn-in. To corroborate the Bayesian inference trees, maximum-likelihood trees were also generated using MEGA software (Kumar et al., 2018). The resulting phylogenetic trees were visualized and annotated using FigTree v1.4.4,^4^ Adobe Illustrator 2022 v26.0.1, and iTOL v6 (Letunic and Bork, 2021).

## 3 Results

### 3.1 Overview of marmot blood virome

**Figure 1:**
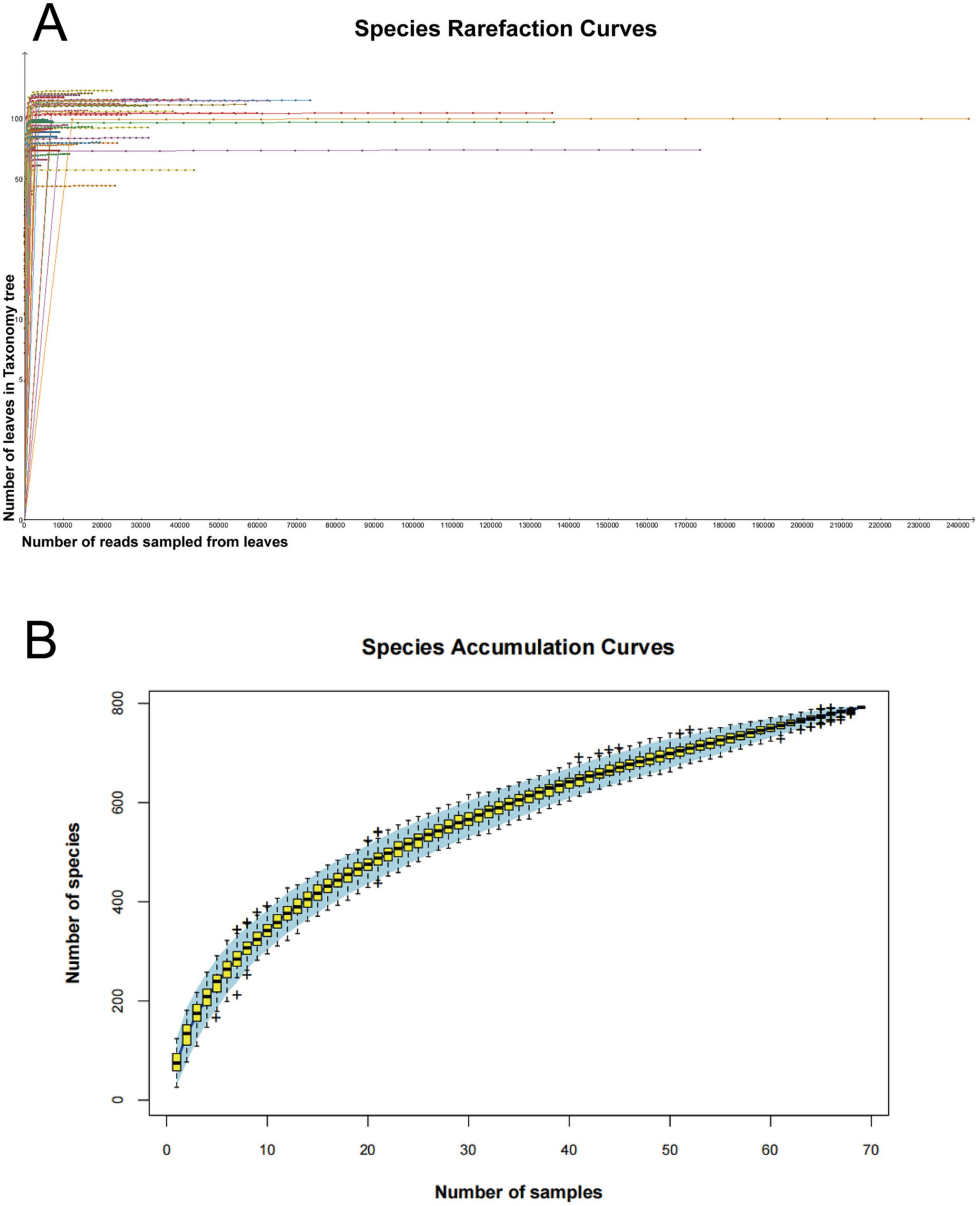
Viral diversity across 70 sequencing libraries. **(A)** Rarefaction curve of viral species generated with MEGAN v6.22.2 (log-transformed *y*-axis). **(B)** Species accumulation curve for the marmot blood virome. Error bars represent the data range; the shaded blue band indicates the 95% confidence interval.

The analysis of species richness in all marmot blood sediment samples, based on species-level rarefaction curves and accumulation curves, revealed the diversity characteristics of the viral community. Detailed library statistics are provided in Supplementary Table S1. In most of the 70 libraries, the number of observed virus families stabilized when the viral sequence count reached a specific threshold, indicating that the sequencing depth in the vast majority of the libraries was sufficient to represent the distribution characteristics of virus families in the blood samples of marmots. Furthermore, the sequencing data were deemed reasonable and reliable (Figure 1A). Although there is significant heterogeneity in viral taxonomic composition among different samples, the viral species accumulation curve has approached saturation, suggesting that the viral community in marmots has been adequately sampled with good representativeness (Figure 1B). The accumulation curve further suggests that there are likely over 800 distinct viral species present across the 70 libraries.

Taxonomic analysis identified 18 specific and functionally complete viral genome sequences obtained through *de novo* assembly and reference alignment, including four known DNA viruses and one RNA virus capable of infecting vertebrates. These viruses were classified as follows: *Anelloviridae* (*n* = 14, one of which exhibited a complete circular genome), *Microviridae* (*n* = 1), *Parvoviridae* (*n* = 1), *Polyomaviridae* (*n* = 1), and *Flaviviridae* (*n* = 1).

### 3.2 Viral diversity and comparison in the viral communities

**Figure 2:**
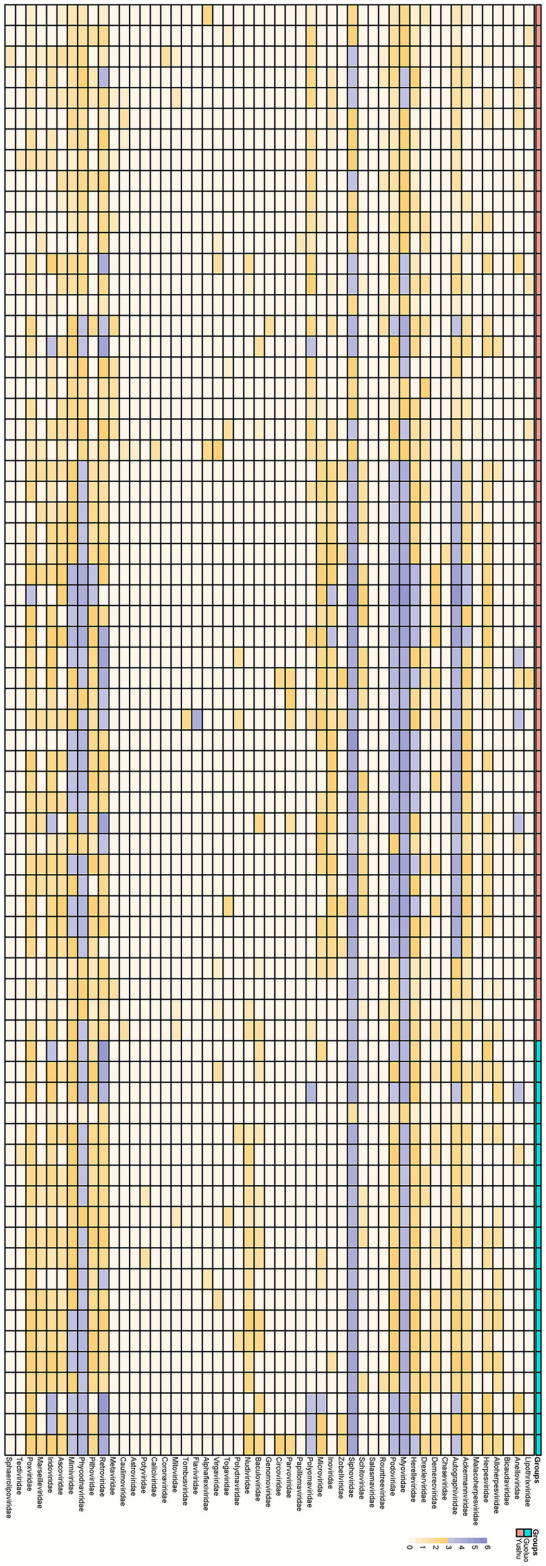
Heatmap of viral family abundance across sequencing pools. Read counts for each viral family are normalized and displayed on a log_10_ scale. Viral taxonomic classifications (types, families, and groups) are color-coded according to the legend.

The heatmap (Figure 2) illustrates the relative abundance or distribution of different viral families in two regions (Guoluo and Yushu). The 70 libraries include various viral families, such as *Lipothrixviridae*, *Anelloviridae*, *Herpesviridae*, and *Flaviviridae*, encompassing both DNA and RNA viruses, and spanning diverse host ranges (e.g., vertebrates, invertebrates, etc.). The distribution of these viral families was compared between the two regions, Guoluo and Yushu. In terms of DNA viruses, *Herpesviridae* is widely distributed in both Yushu and Guoluo, with higher abundance in Yushu. *Polyomaviridae* is primarily distributed in Yushu, with lower abundance in Guoluo. *Parvoviridae*, *Circoviridae*, and *Poxviridae* are present in both regions, although their overall abundance is relatively low. Regarding RNA viruses, *Flaviviridae* exhibits extremely high abundance in certain Yushu samples, while *Coronaviridae* is predominantly distributed in Yushu, with no detection in Guoluo.

**Figure 3:**
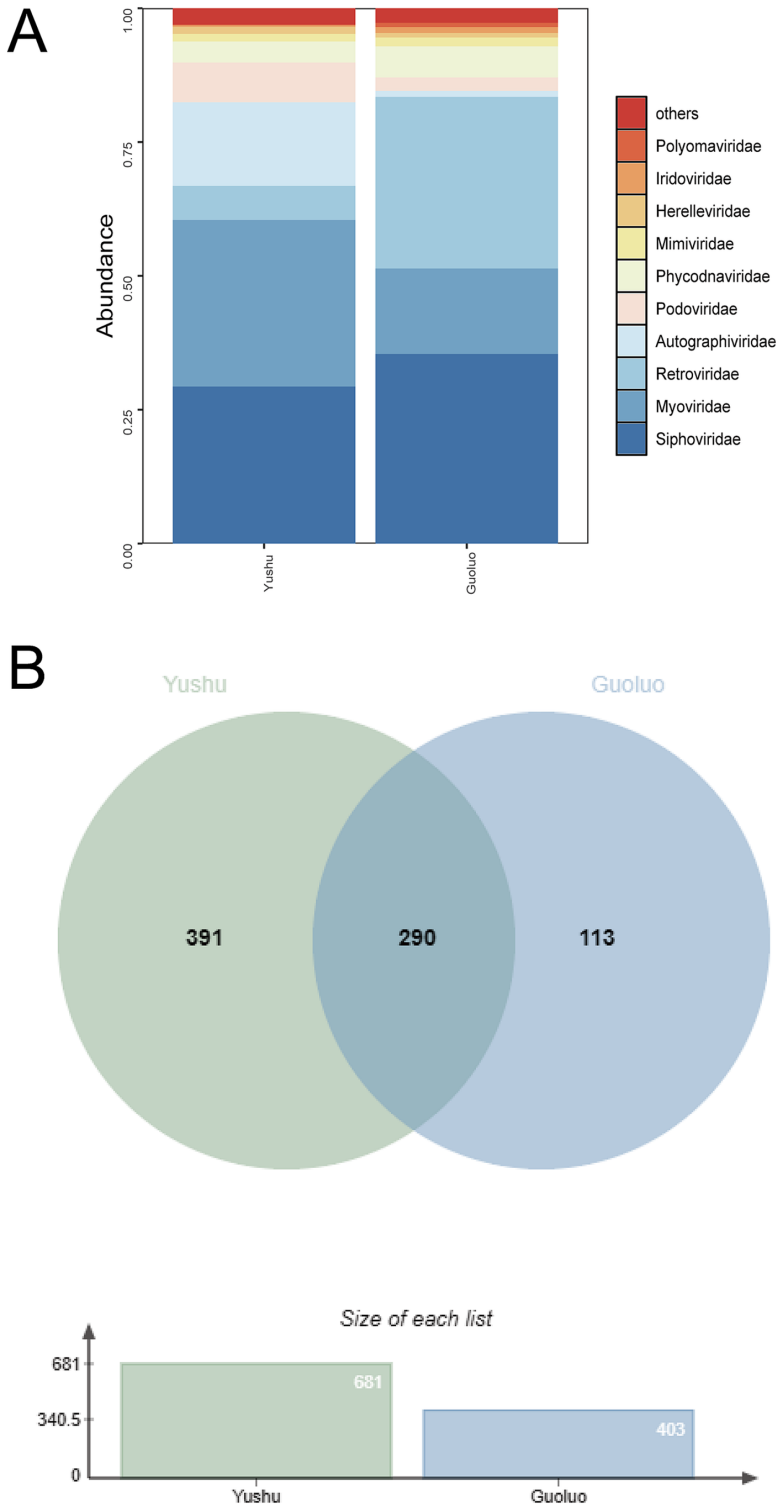
Taxonomic analysis of viral reads at the family and species levels. **(A)** Relative abundance of viral families, showing taxonomic classification and proportional distribution. **(B)** Shared and unique viral species between the two sample groups, visualized by Venn diagram.

In the Yushu region, analysis of viral family relative abundance revealed that *Siphoviridae* and *Myoviridae* dominated the community composition, exhibiting the highest proportions (Figure 3A). Host-based classification further confirmed that bacteriophage-associated viruses constituted the predominant fraction of the virome in Yushu, exceeding their relative contribution in Guoluo. In contrast, the viral community in Guoluo was predominantly characterized by *Retroviridae*, with its abundance significantly surpassing that of *Myoviridae*. At the species level, the two regions shared 290 viral species, while the proportion of species unique to Yushu (57.4%, 391/681) markedly exceeded that of Guoluo (28.0%, 113/403). Collectively, these patterns indicate that the Yushu virome is characterized by greater taxonomic richness and a stronger bacteriophage dominance than that of Guoluo (Figure 3B).

**Figure 4:**
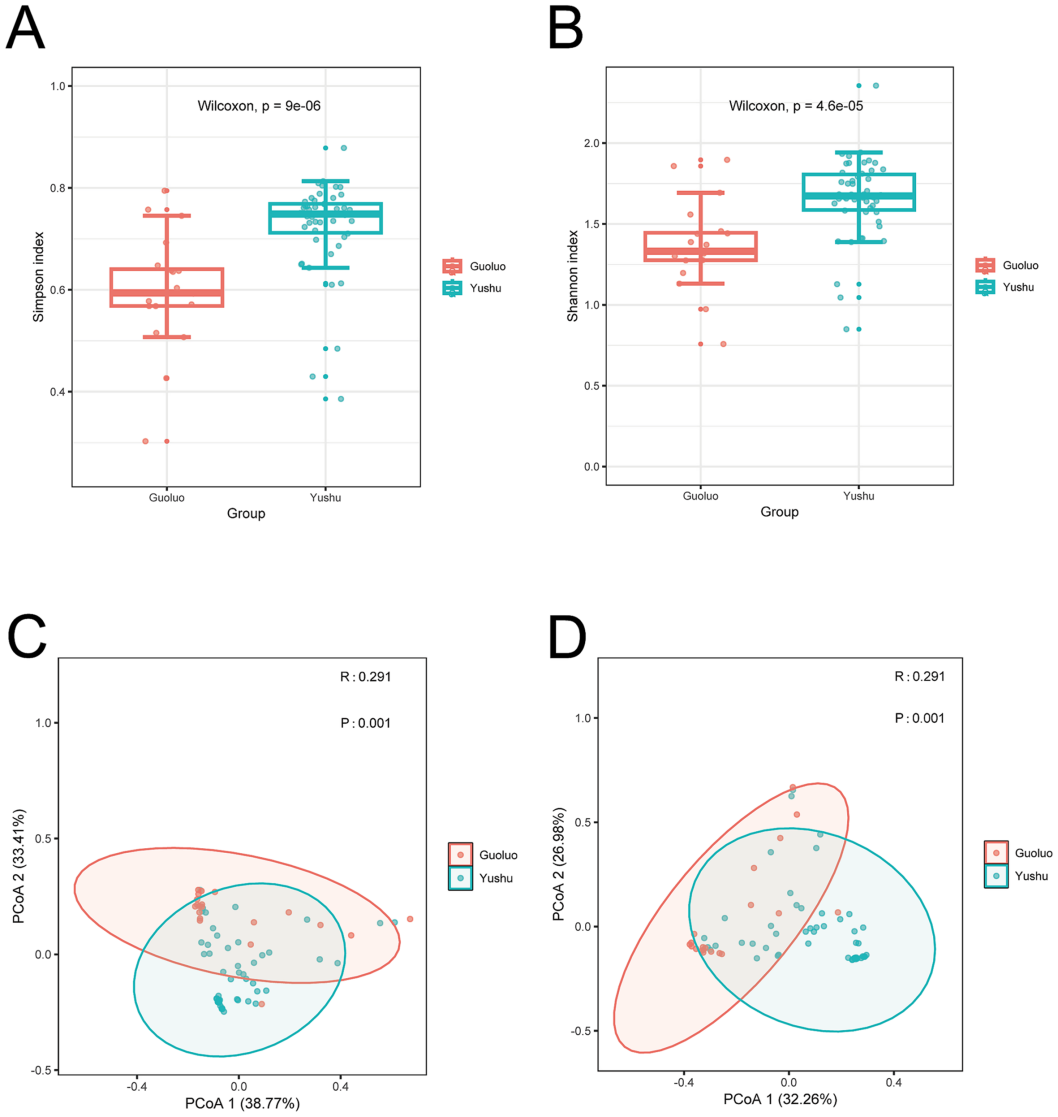
Diversity of viral communities between Yushu and Guoluo. **(A,B)** Comparison of viral alpha diversity, normalized using MEGAN and measured using the Shannon index based on viral abundance at the family level. The *p*-value was calculated using the Wilcoxon test. Horizontal bars within the boxes represent medians, while the tops and bottoms of the boxes indicate the 75th and 25th percentiles, respectively. **(C,D)** Principal coordinate analysis (PCoA) based on Bray–Curtis and Jaccard distances, illustrating viral beta diversity normalized using MEGAN at the family level. A *p*-value <0.05 was considered statistically significant.

To systematically compare the viral community differences, the Shannon index and Simpson index were used to assess alpha diversity and further analyze the differences in viral composition between Yushu and Guoluo. The results showed significant differences in viral communities between the two regions (*p* < 0.01), as shown in Figures 4A,B. Specifically, Yushu exhibited significantly higher alpha diversity compared to Guoluo. Principal coordinate analysis (PcoA) based on Bray–Curtis (Figure 4C) and Jaccard distances (Figure 4D) revealed statistically significant differences in the viral community composition at the family level between the two regions based on beta diversity (*p* = 0.01).

### 3.3 Identification of *Anelloviridae*

*Anelloviridae* is a family of single-stranded circular DNA viruses with a genome length of approximately 2.8–3.9 kb. The virus is non-enveloped, and its capsid exhibits an icosahedral symmetrical structure (Biagini, 2009). The genome contains several open reading frames (ORFs), with ORF1 encoding the capsid protein, and ORF2/ORF3 involved in viral replication and regulation.

*Anelloviridae* viruses are known to infect vertebrates, including humans, other mammals, and birds (Yan et al., 2024). In humans, the virus is primarily transmitted through blood products, vertical transmission from mother to child, and close contact (e.g., saliva, feces) (Kaczorowska and van der Hoek, 2020). Most infections are asymptomatic, but in immunocompromised individuals (such as organ transplant recipients and HIV-infected individuals), increased viral load may be associated with risks such as graft rejection, hepatitis, or cancer, although causality has not been conclusively established (Spandole et al., 2015).

**Figure 5:**
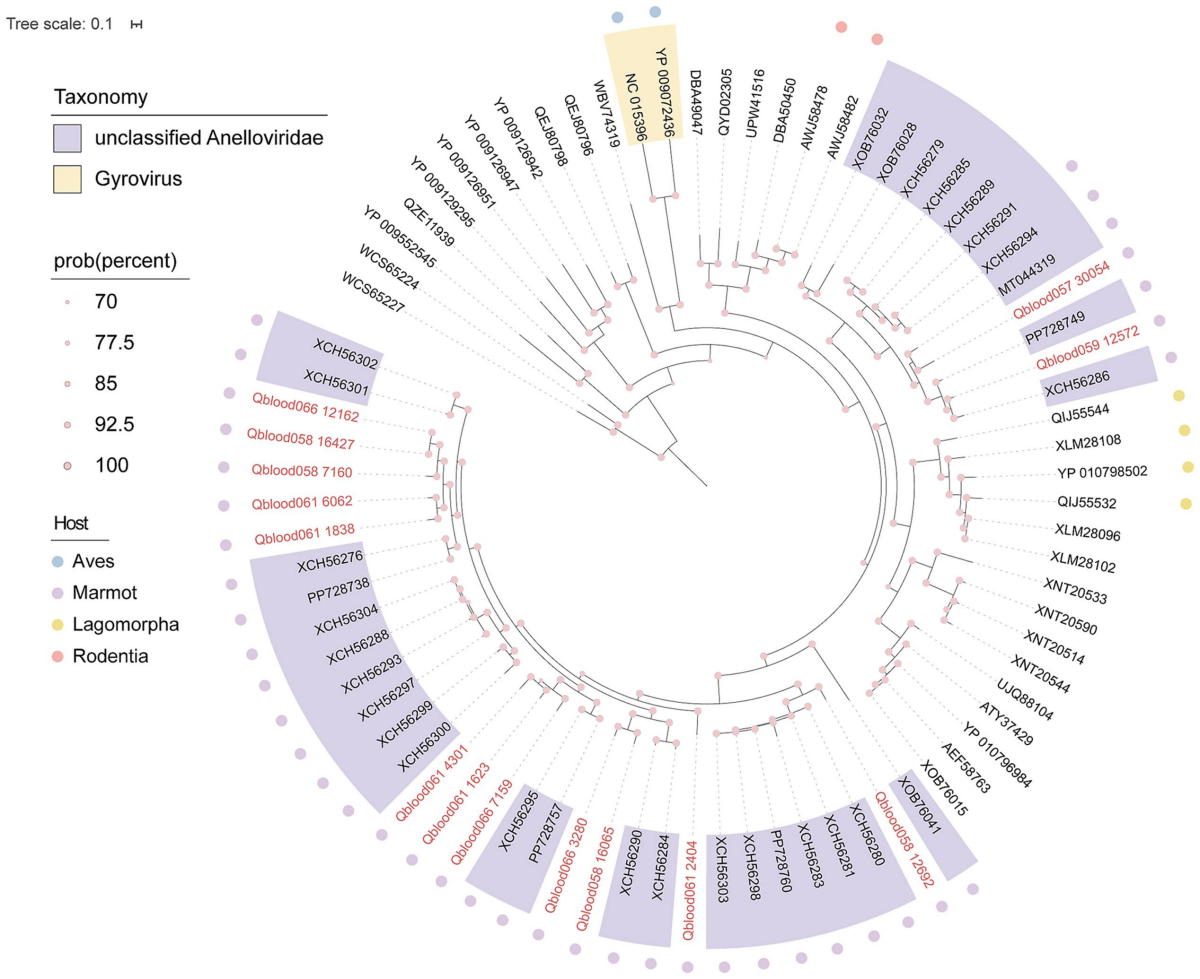
Phylogenetic analysis of *Anelloviridae* based on ORF1 protein sequences. The sequence identified in this study is highlighted in pink. Relevant taxonomic and feature annotations are provided in the figure legend.

Through sequence assembly and functional annotation of high-throughput sequencing data, this study identified a total of 14 *Anelloviridae* viral sequences, one of which (Qblood059_12572) exhibited a complete circular genome structure. To investigate their phylogenetic relationships, the ORF1 genes of the 14 sequences were aligned with reference sequences downloaded from the GenBank database and a Bayesian phylogenetic tree was constructed (Figure 5). The results showed that all 14 sequences clustered with known viral sequences isolated from marmot (*Marmota* spp.) feces or tissues. BLASTx comparison analysis revealed that the amino acid similarity between these sequences and the reference sequences ranged from 69 to 94%, and they were all classified as *unclassified Anelloviridae*. Notably, some sequences exhibited unique clustering patterns in the phylogenetic tree. Qblood066_12162, Qblood058_16427, Qblood058_7160, Qblood061_6062, and Qblood061_1838 formed an independent branch, Qblood061_4301, Qblood061_1623, and Qblood066_7159 formed another independent branch, and Qblood066_3280 and Qblood058_16065 also showed independent clustering. These branches may represent new genera or species within the *Anelloviridae* family. Qblood058_12692 formed a separate branch in the phylogenetic tree, suggesting it may belong to a new genus-level taxon.

Based on phylogenetic analysis of the ORF1 sequences, all marmot anelloviruses identified in this study were not assigned to any known genus and are currently designated as unclassified species within the family *Anelloviridae*. Phylogenetic analysis further indicated that the host range of *Anelloviridae* is broad, encompassing various vertebrate species. However, as a potential host, the diversity of *Anelloviridae* viruses within marmots has yet to be fully explored, suggesting that this species could serve as an important model for studies on the evolution and host adaptation of *Anelloviridae* viruses.

**Figure 6:**
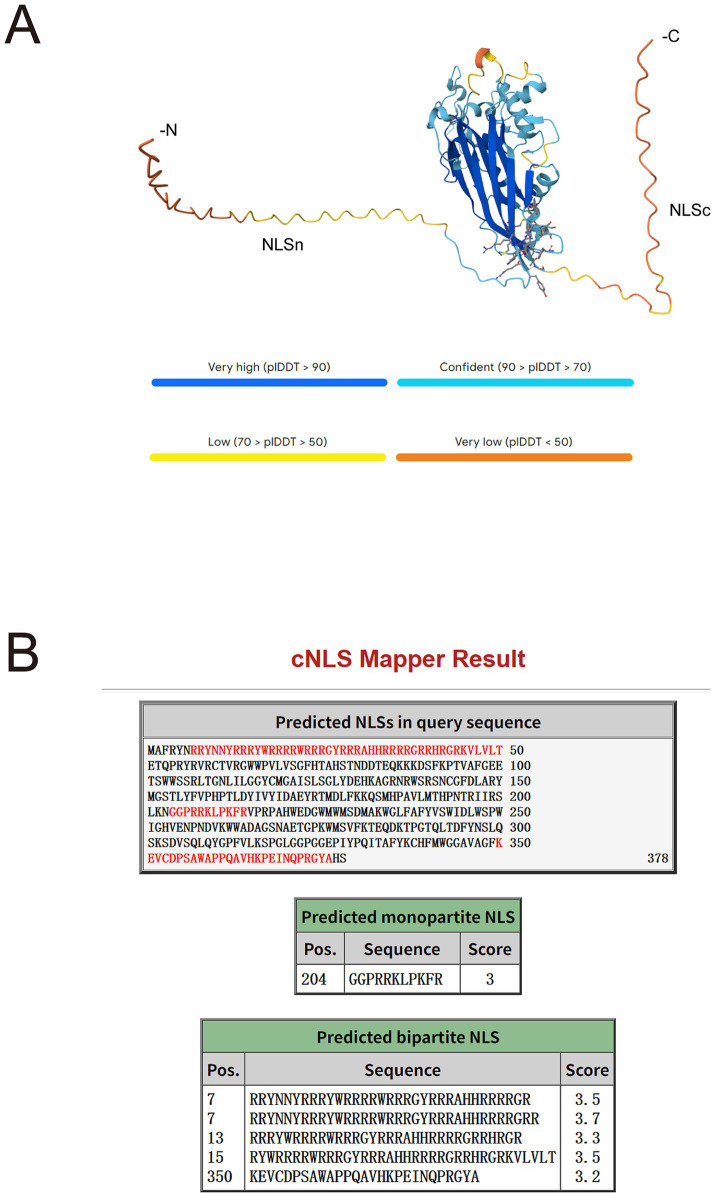
Prediction of NLSs in the Anelloviridae ORF1 protein. **(A)** AlphaFold3 model of ORF1. The top ranked prediction is shown; coloured by pLDDT score of estimated confidence: very high (pLDDT >90) in dark blue, confident (90 >pLDDT >70) in light blue, low (70 >pLDDT >50) in yellow, and very low (pLDDT <50) in orange. **(B)** Mapping of putative NLSs by cNLS Mapper analysis. Top: Schematic representation of the ORF1 protein sequence (single-letter amino acid code), with predicted NLS motifs highlighted in red. Bottom: Detailed view of each predicted NLS, indicating its position, sequence, and corresponding cNLS Mapper score.

*Anelloviridae* capsids exhibit broad heterogeneity in amino acid length due to variable projection domains, despite a conserved jelly-roll fold (Butkovic et al., 2023). Intriguingly, while all *Anelloviridae* capsids encode an N-terminal arginine-rich motif (ARM) that functions as a non-classical nuclear localization signal (NLS), only those with large projection domains have evolved a unique classical nuclear localization signal (cNLS) at their C-terminus (Petersen et al., 2025). “NLSs” refers broadly to all types of nuclear localization signals, while “cNLS” specifically denotes classical nuclear localization signals, which are transported into the nucleus via the Importin *α*/β1 pathway (Hoad et al., 2026). To further investigate this issue, we structurally predicted the three-dimensional structure of the ORF1 protein encoded by one of the newly identified anelloviruses (Qblood059_12572) and utilized the cNLS Mapper to identify putative cNLSs. The analysis predicted a typical jelly-roll fold in the central region of Qblood059_12572 ORF1 (residues 59–342), accompanied by a small projection domain (Figure 6A). On the other hand, the N- and C-termini of the protein are predicted to be largely unstructured and contain multiple putative NLSs with low scores (Figure 6B), indicating that they are likely non-functional as cNLSs. Therefore, Qblood059_12572 encodes an ORF1 protein with a jelly-roll fold and a very short projection domain, and lacks a C-terminal NLS, consistent with recent reports (Butkovic et al., 2023; Petersen et al., 2025).

### 3.4 Phylogenetic analysis of Tick-borne encephalitis virus

The *Flaviviridae* family comprises single-stranded, positive-sense RNA viruses (+ssRNA) with a genome length of approximately 10–11 kilobases (kb). These viruses are enveloped, and their capsid proteins exhibit icosahedral symmetry (Pierson and Diamond, 2020). Tick-borne encephalitis virus (TBEV), a member of the *Orthoflavivirus* genus within the *Flaviviridae* family, is primarily transmitted through the bite of hard ticks, such as *Ixodes ricinus* and *Ixodes persulcatus* (Yoshii, 2019). TBEV is predominantly distributed across forested and grassland regions of Europe, Northern Asia, and Eastern Asia, with significant epidemiological prevalence in Russia, Central Europe, and Northern Europe (Ruzek et al., 2019).

**Figure 7:**
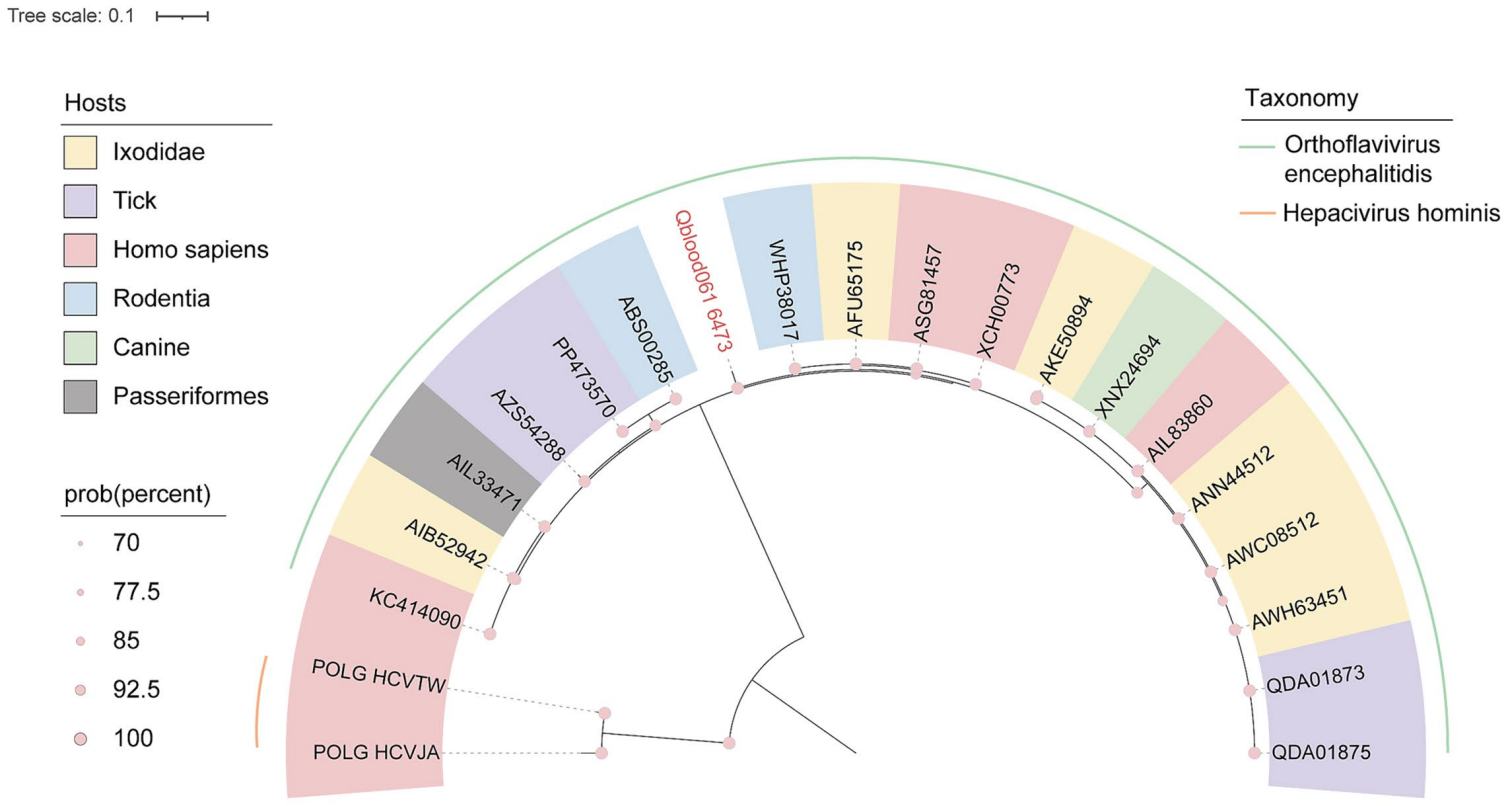
Phylogenetic relationship of Tick-borne encephalitis virus. Phylogenetic tree based on RdRp amino acid sequences of the *Flaviridae* viruses. The pink name indicates the sequence obtained in this study. See legend for relevant labeling.

In this study, a viral sequence (Qblood061_6473) with a length of 6,550 bp was identified, though its polyprotein-coding region was incomplete. BLASTx alignment analysis revealed a 94.31% amino acid similarity to a reference sequence isolated from human brain tissue (GenBank Accession No. KC414090). Phylogenetic tree analysis demonstrated that Qblood061_6473 clustered with members of the *Orthoflavivirus* genus, which are known to infect a diverse range of hosts (Figure 7). It is noteworthy that this is the first time a viral sequence belonging to the *Orthoflavivirus* genus has been identified in the blood of marmots, suggesting that marmots may be potential hosts for TBEV.

### 3.5 Phylogenetic analysis of *Parvoviridae*

The family *Parvoviridae* comprises small, non-enveloped viruses with a single-stranded DNA genome, approximately 4–6 kb in length, encapsulated within an icosahedral capsid (Decaro and Buonavoglia, 2012). This viral family exhibits a broad host range, infecting mammals, birds, and insects, and is associated with a variety of diseases (Dai et al., 2022). For instance, Canine parvovirus causes severe enteric disease in dogs (Nandi et al., 2010), Porcine parvovirus is linked to reproductive failure and abortion in swine (Streck and Truyen, 2020), and Human parvovirus B19 is implicated in conditions such as erythema infectiosum (fifth disease) in children and arthritis in adults (Dittmer et al., 2024).

**Figure 8:**
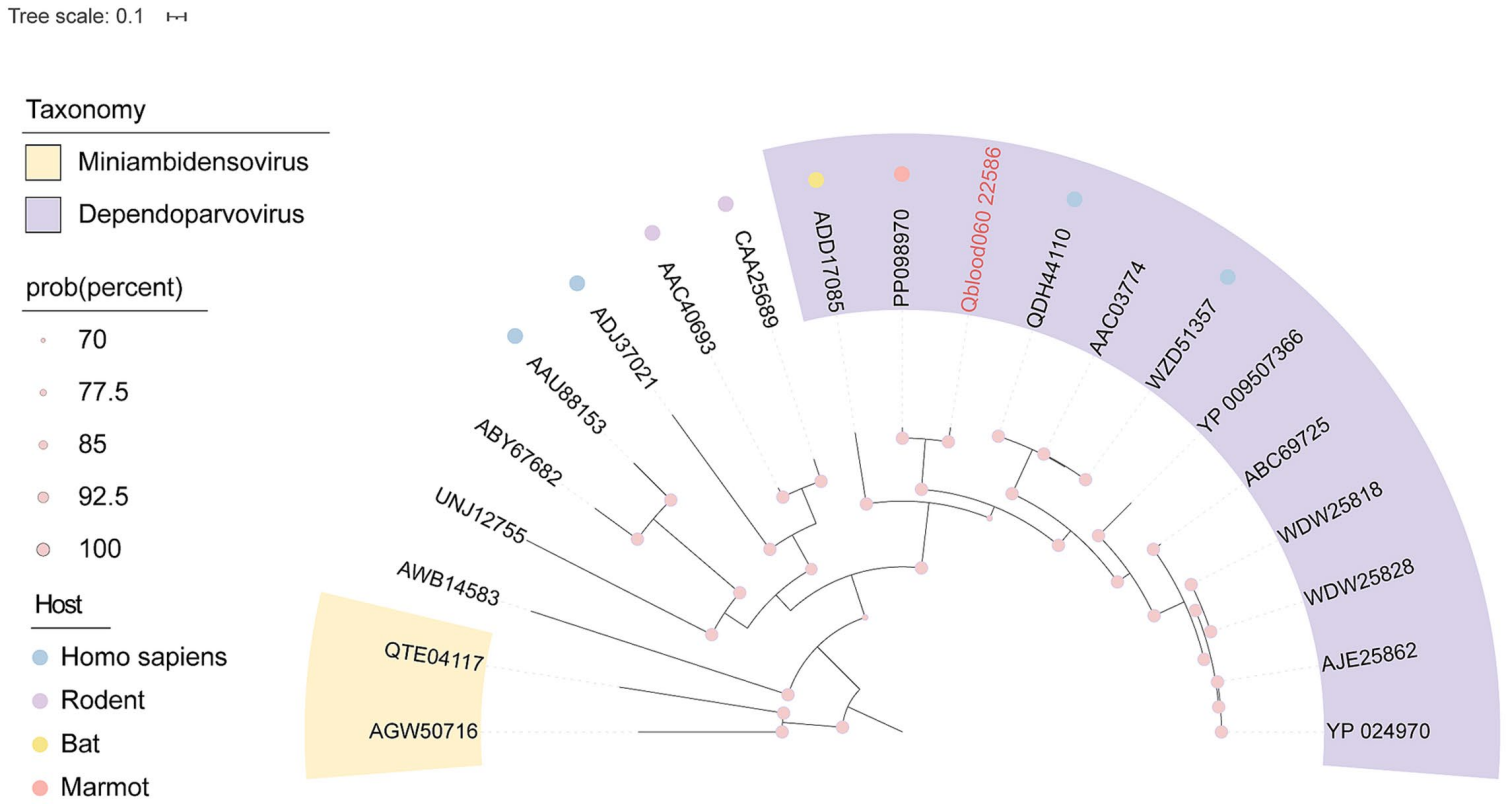
Phylogenetic relationship of *Parvoviridae*. Phylogenetic tree based on rep protein sequences. The pink name indicates the sequence obtained in this study. Taxonomy and hosts are annotated with corresponding colors, as indicated in the color legend.

In this study, a novel viral sequence, designated as Qblood060_22586, was identified through high-throughput sequencing and bioinformatics analysis. The viral genome has a total length of 4,599 bp and contains complete replication-associated protein (rep) and capsid protein (cap) genes, indicating a fully intact viral genome structure. BLASTx alignment analysis revealed that the Rep protein exhibits high similarity to viruses within the genus *Dependoparvovirus*. The highest similarity (88.63%) was observed with a Dependoparvovirus sequence derived from marmot tissue (GenBank Accession No. PP098970), and it clustered with this sequence in phylogenetic analysis (Figure 8). Additionally, the Rep protein showed 57.36% similarity to a *Dependoparvovirus* sequence from caprine. These results suggest that the virus Qblood060_22586 likely belongs to the genus *Dependoparvovirus*, with marmots being a potential host.

### 3.6 Phylogenetic analysis of *Polyomaviridae*

**Figure 9:**
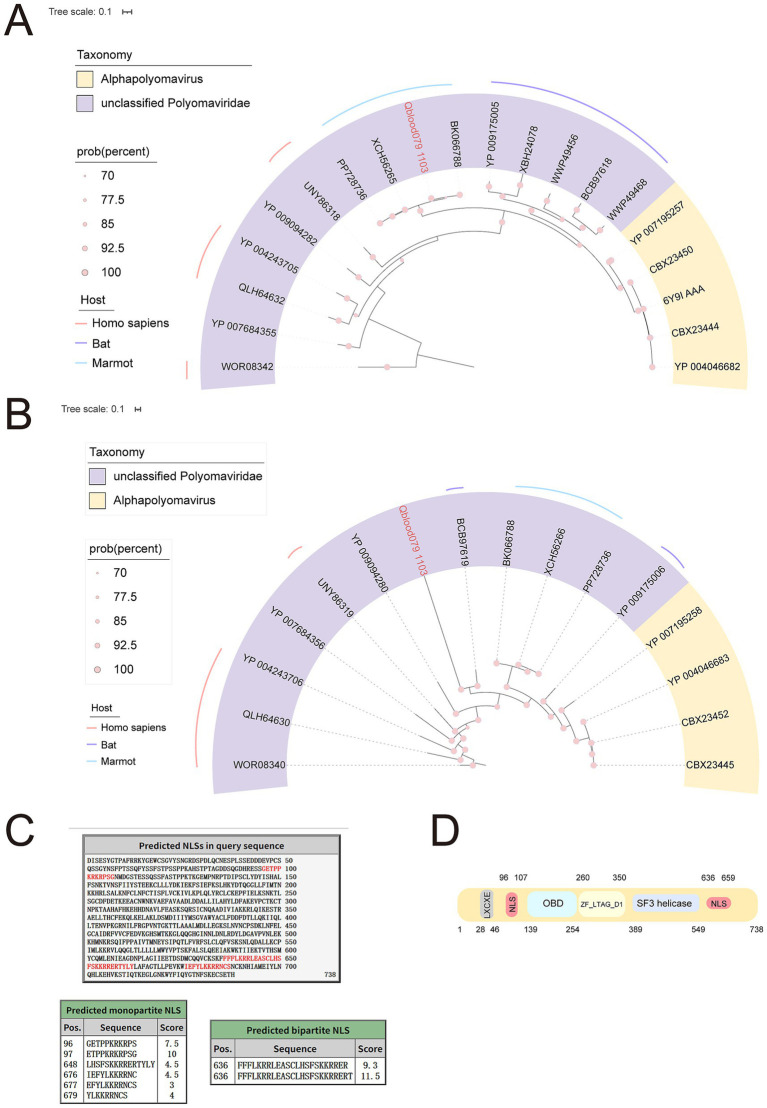
Phylogenetic and structural analysis of a novel polyomavirus. **(A)** Phylogenetic tree inferred from VP1 protein sequences of *Polyomaviridae*. The sequence obtained in this study is highlighted in pink. Taxonomy and hosts are annotated with corresponding colors, as indicated in the legend. **(B)** Phylogenetic tree based on LTA protein sequences. **(C)** Predicted nuclear localization signal (NLS) positions within the LTA domain. Key functional motifs are annotated. **(D)** A schematic representation of the novel virus LTA domains, using the SV40 LTA aa positions as a reference.

*Polyomaviridae* is a small family of double-stranded circular DNA viruses, typically characterized by non-enveloped viral particles with a diameter of about 40–50 nanometers. Members of the family *Polyomaviridae* transcribe distinct early and late precursor mRNAs. The early transcript undergoes alternative splicing to generate the small, middle (when present), and large T antigens, while the late transcript encodes the structural proteins (Wiley et al., 1993). The *Polyomaviridae* family includes more than a dozen human-infecting species, notably John Cunningham polyomavirus (JCPyV), BK polyomavirus (BKPyV), Merkel cell polyomavirus (MCPyV), and Trichodysplasia spinulosa polyomavirus (TSPyV), which are clearly linked to human disease. Several avian polyomaviruses also exhibit strong pathogenic potential (DeCaprio and Garcea, 2013). Polyomaviridae can cause various diseases, including tumors and immunosuppression (Loutfy et al., 2017). The host range is broad, including humans, monkeys, mice, birds, and others. In this study, a novel polyomavirus, tentatively designated as Qblood079_1103, was identified using high-throughput sequencing combined with comprehensive bioinformatic analysis. The complete viral genome spans 5,188 bp and encodes a full-length capsid protein VP1, confirming its structural integrity as a member of the *Polyomaviridae* family. Comparative analysis using BLASTx revealed that the VP1 protein shares significant sequence homology with unclassified polyomaviruses, demonstrating the highest identity (86.72%) with a reference polyomavirus strain isolated from the skin tissue of a marmot (GenBank Accession No. BK066788). Phylogenetic reconstruction based on VP1 protein sequences placed the newly identified virus within a monophyletic clade alongside the marmot-derived polyomavirus, indicating a close evolutionary relationship (Figure 9A). Concurrently, we also constructed a phylogenetic tree for the large T antigen (LTA) protein (Figure 9B). The results closely parallel those obtained with the VP1 protein. The LTA is a multifunctional viral protein that orchestrates viral DNA replication and host cell cycle modulation. It contains several conserved domains, including the J domain, HPDKGG motif, LXCXE motif (for RB binding), the origin-binding domain (OBD), and the SF3 helicase domain (Topalis et al., 2013). These activities depend on its nuclear localization. Accordingly, a cNLS was first identified in SV40 LTA between the LXCXE motif and the OBD (Kalderon et al., 1984).

Intriguingly, comparative analyses show that all mammalian-infecting polyomaviruses encode an LTA bearing an NLS at this conserved position (Cross et al., 2024), whereas non-mammalian polyomaviruses encode NLSs elsewhere (Hoad et al., 2026). Therefore, identification of LTA functional domains and mapping the NLS in the newly identified virus may provide valuable insights into its host origin and evolutionary relationships. We therefore identified the principal functional motifs and domains within the LTA from Qblood079_1103. Our analysis indicates that this protein retains all major LTA functional regions and contains a putative bipartite cNLS located between the LXCXE and OBD motifs, supporting its classification as a mammalian polyomavirus (Figures 9C,D). Additionally, an additional NLS is located downstream of the SF3 helicase domain (Figures 9C,D).

## 4 Discussion

This study aimed to elucidate the viral diversity in the blood of marmots in the Qinghai–Tibet Plateau region using high-throughput sequencing and bioinformatics analysis, with the goal of assessing their role as potential viral reservoirs and exploring the potential implications of these viruses for wildlife and human health. To this end, we collected 70 marmot blood samples from Yushu and Guoluo Prefectures in Qinghai Province and conducted a comprehensive analysis of the viral communities in these samples using advanced molecular biology and bioinformatics techniques. Our research not only expands the understanding of the marmot virome but also provides critical data for understanding viral transmission dynamics in wildlife and their potential public health risks.

Through high-throughput sequencing and bioinformatics tools, we identified a wide range of viral families, including *Anelloviridae*, *Flaviviridae*, *Parvoviridae*, and *Polyomaviridae*, among others (Figure 2). Our findings not only enhance the current understanding of viral ecology in marmots but also highlight the potential role of these rodents as reservoirs for zoonotic viruses, which has significant implications for wildlife conservation and public health.

The observed variation in the relative proportions of bacteriophages and vertebrate-associated viruses between Yushu and Guoluo likely reflects differences in local ecological conditions and host availability (Figure 3). The higher dominance of bacteriophages in Yushu suggests a viral community structure more strongly shaped by bacterial hosts, potentially associated with differences in microbial community composition or environmental factors. In contrast, the relatively increased proportion of vertebrate-associated viruses in Guoluo may indicate stronger inputs from vertebrate hosts or host-derived materials, possibly linked to animal density or human–animal interactions. Overall, these findings highlight spatial heterogeneity in viral community structure and emphasize the influence of local host ecology on shaping virome composition.

The identification of *Anelloviridae* in marmot blood samples is particularly noteworthy. While Anelloviridae viruses are known to infect diverse vertebrates including humans (De Souza et al., 2018), this study provides the first documented evidence of their presence in marmot serum. Phylogenetic analysis revealed that the *Anelloviridae* sequences identified in this study clustered with known sequences from marmot feces and tissues (Figure 5), suggesting that marmots may serve as natural hosts for these viruses. The high genetic diversity observed among the *Anelloviridae* sequences further underscores the potential role of marmots in the evolution and host adaptation of these viruses. Future studies should explore the pathogenicity of these viruses in marmots and their potential for cross-species transmission. This study confirms that the ORF1 protein encoded by Qblood059_12572 possesses a typical jelly-roll fold and an extremely short projection domain, while lacking a functional C-terminal nuclear localization signal (Figure 6). These structural characteristics are fully consistent with recent descriptions of anelloviruses with small projection domains (Petersen et al., 2025). It is worth noting that the predicted C-terminal region exhibits low confidence (pLDDT) in the AlphaFold model. Therefore, inferences about the absence of a functional nuclear localization signal in this region based on the current model should be made with caution and require experimental validation.

The detection of Tick-borne encephalitis virus (TBEV) in marmot blood samples is another significant finding. TBEV, a member of the *Flaviviridae* family, is a highly pathogenic virus that can cause severe neurological disorders in humans (Blom et al., 2018). The identification of a TBEV sequence with high similarity to a human-derived reference sequence suggests that marmots may play a role in the transmission cycle of this virus (Figure 7). This finding aligns with previous studies that have implicated marmots as potential hosts for TBEV, particularly in regions where they coexist with human populations (Dai et al., 2018). Although the TBEV sequences we identified show high similarity to human pathogenic strains, the incomplete genome and lack of functional data mean we cannot assess their actual infectivity and transmission capacity. Therefore, considering marmots as part of the transmission cycle of this virus remains a hypothesis requiring further validation.

In addition to *Anelloviridae* and *Flaviviridae*, we also identified novel sequences belonging to the *Parvoviridae* and *Polyomaviridae* families. The *Parvoviridae* sequence, designated as Qblood060_22586, exhibited high similarity to a *Dependoparvovirus* sequence derived from marmot tissue, suggesting that marmots may be a natural host for this virus. Similarly, the *Polyomaviridae* sequence, Qblood079_1103, showed close phylogenetic relationships with a marmot-derived polyomavirus (GenBank Accession No. BK066788), further supporting the idea that marmots harbor a diverse array of viruses. These findings contribute to the growing body of evidence that marmots are important reservoirs for a variety of viral pathogens. Meanwhile, we also analyzed the presence of functional domains within the LTA protein (Figure 9), which might help clarify the relationship between the newly identified isolate and other species. For the novel polyomavirus identified in this study, both the predicted nuclear localization function of its LTA protein and its actual host range remain unknown. Future studies should clone the LTA gene of this virus and express it in mammalian cells, using immunofluorescence to directly observe its subcellular localization, thereby providing experimental evidence for its function.

It is critical to note that the all main LTA functional domains could be predicted in the LTA encoded by Qblood079_1103, including a strong putative bipartite cNLS located between the LXCXE motif and the OBD, similarly to all LTA from mammalian-infecting polyomaviruses studied so far (Cross et al., 2024). Intriguingly, a bipartite NLS was also predicted downstream of the SF3 helicase domain, in a similar position to NLSs recently characterized in the LTA from fish infecting polyomaviruses (Hoad et al., 2026). In the present study, the predicted NLS is located downstream of the SF3 helicase domain. This observation aligns with the hypothesis that the position and composition of NLS motifs in large T-like proteins are evolutionarily plastic, capable of shifting among the N-terminal, central, and C-terminal regions.

The comparative analysis of viral communities between Yushu and Guoluo revealed significant differences in viral diversity and abundance. Yushu exhibited a higher diversity of viral families, including *Herpesviridae*, *Polyomaviridae*, and *Flaviviridae*, compared to Guoluo. This regional variation in viral communities may be influenced by ecological factors such as habitat type, climate, and human activity. The higher viral diversity in Yushu could also reflect differences in marmot population density or the presence of additional reservoir species. Further studies are needed to elucidate the factors driving these regional differences and their implications for viral transmission dynamics. It should be noted that the observed regional variations in the virome, while potentially associated with habitat types, may also be influenced by other unmeasured confounding factors, such as host age structure, population density, immune status, and differences in ectoparasite communities. Future studies should systematically collect data on these covariates to more comprehensively elucidate the key drivers underlying the geographical variation of the virome.

One limitation of this study is the reliance on blood samples, which may not capture the full spectrum of viruses present in marmots. Future research should include other sample types, such as feces, urine, and tissue samples, to provide a more comprehensive picture of the marmot virome. It is important to emphasize that the viral sequences detected in this study only demonstrate the presence of viral nucleic acids. Confirming active replication and pathogenicity of these viruses in marmots requires further validation through future studies involving virus isolation, targeted serological surveys (e.g., IgM detection), or experimental infection approaches. Importantly, due to the limited availability of samples, we were unable to perform experimental validation of the identified viral sequences in this study. While these high-quality viral contigs provide valuable clues for future investigations, their biological relevance and authenticity require confirmation through independent experimental approaches. Further studies are needed to determine the potential impact of these viruses on marmot health and their capacity for cross-species transmission.

In conclusion, this study provides valuable insights into the viral diversity of marmots in the Qinghai–Tibet Plateau region. The identification of novel viruses and the detection of known pathogens such as TBEV highlight that marmots may serve as potential reservoirs for zoonotic viruses. These findings have important implications for wildlife conservation and public health, particularly in regions where marmots live in close proximity to human populations. Enhanced surveillance and further research are needed to better understand the role of marmots in viral transmission and to develop effective strategies for mitigating the risk of zoonotic disease outbreaks.

## Data Availability

The viral metagenomic data utilized to corroborate the findings of this study has been submitted and deposited at the National Genomics Data Center. The quality-filtered sequencing data have been deposited in the Sequence Read Archive (SRA) under BioProject accession PRJCA035278 and BioSample accessions SAMC4593464-SAMC4593533. All newly identified genes have been registered with and assigned sequence identifiers (C_AA105711.1-C_AA105727.1) by the National Genomics Data Center. These data are publicly available without any access restrictions.

## Footnotes

1
http://nls-mapper.iab.keio.ac.jp/

2
https://prosite.expasy.org/

3
http://elm.eu.org/

4
http://tree.bio.ed.ac.uk/software/figtree/
