# Supplementary material for: Characterization of viral diversity in wild marmot blood from the Qinghai–Tibet Plateau
Source: Front Microbiol. 2026 Feb 6;17:1668126. doi: 10.3389/fmicb.2026.1668126 (PMC12920470; doi:10.3389/fmicb.2026.1668126)
Supplement: Supplementary file 1 [file Table_1.DOCX]

**Table S1. Sampling information** **of marmots**

| Pool name | number of reads | number of viral reads |
| --- | --- | --- |
| Qblood027 | 473,178 | 1,169 |
| Qblood028 | 1,356,988 | 4,738 |
| Qblood029 | 1,511,014 | 6,492 |
| Qblood030 | 3,000,502 | 17,115 |
| Qblood031 | 1,422,204 | 10,166 |
| Qblood032 | 1,063,152 | 2,920 |
| Qblood033 | 1,468,492 | 4,899 |
| Qblood034 | 870,268 | 1,825 |
| Qblood035 | 1,256,388 | 5,991 |
| Qblood036 | 1,195,640 | 2,749 |
| Qblood037 | 1,153,948 | 3,730 |
| Qblood038 | 1,319,162 | 2,690 |
| Qblood039 | 8,060,090 | 28,249 |
| Qblood040 | 1,367,402 | 9,090 |
| Qblood041 | 52,772 | 560 |
| Qblood042 | 6,416,778 | 50,338 |
| Qblood043 | 29,779,336 | 73,652 |
| Qblood044 | 1,534,408 | 7,860 |
| Qblood045 | 748,196 | 3,128 |
| Qblood046 | 918,980 | 5,451 |
| Qblood047 | 1,534,212 | 10,237 |
| Qblood048 | 854,326 | 4,171 |
| Qblood049 | 4,894,640 | 54,161 |
| Qblood050 | 6,321,536 | 46,295 |
| Qblood051 | 4,985,600 | 46,521 |
| Qblood052 | 7,470,772 | 81,257 |
| Qblood053 | 11,693,996 | 102,355 |
| Qblood054 | 29,882,748 | 489,602 |
| Qblood055 | 49,723,180 | 859,962 |
| Qblood056 | 12,666,240 | 193,692 |

| Pool name | number of reads | number of viral reads |
| --- | --- | --- |
| Qblood057 | 45,842,178 | 510,694 |
| Qblood058 | 40,761,140 | 148,329 |
| Qblood059 | 25,208,026 | 136,804 |
| Qblood060 | 29,233,192 | 36,703 |
| Qblood061 | 47,306,290 | 92,370 |
| Qblood062 | 18,608,504 | 469,153 |
| Qblood063 | 11,918,644 | 195,859 |
| Qblood064 | 13,375,006 | 211,405 |
| Qblood065 | 7,316,878 | 104,237 |
| Qblood066 | 102,174,676 | 128,442 |
| Qblood067 | 322,640,398 | 19,338 |
| Qblood068 | 13,074,528 | 220,398 |
| Qblood069 | 5,147,246 | 72,422 |
| Qblood070 | 16,381,484 | 257,775 |
| Qblood071 | 10,688,964 | 145,146 |
| Qblood072 | 6,173,102 | 85,228 |
| Qblood073 | 2,252,660 | 8,292 |
| Qblood074 | 2,177,920 | 12,384 |
| Qblood075 | 1,988,348 | 6,424 |
| Qblood076 | 5,519,548 | 30,902 |
| Qblood077 | 166,156,694 | 142,954 |
| Qblood078 | 14,722,964 | 52,961 |
| Qblood079 | 19,031,648 | 113,073 |
| Qblood080 | 151,508 | 618 |
| Qblood081 | 5,662,490 | 40,534 |
| Qblood082 | 5,720,562 | 34,171 |
| Qblood083 | 4,655,060 | 34,235 |
| Qblood084 | 4,081,110 | 26,978 |
| Qblood085 | 3,047,416 | 16,959 |
| Qblood086 | 8,786,764 | 66,548 |

| Pool name | number of reads | number of viral reads |
| --- | --- | --- |
| Qblood087 | 4,731,666 | 45,645 |
| Qblood088 | 3,863,740 | 27,430 |
| Qblood089 | 5,049,234 | 52,212 |
| Qblood090 | 10,501,116 | 96,199 |
| Qblood091 | 7,902,544 | 87,070 |
| Qblood092 | 11,791,318 | 125,311 |
| Qblood093 | 9,053,972 | 62,039 |
| Qblood094 | 114,405,068 | 350,057 |
| Qblood095 | 57,503,120 | 224,502 |
| Qblood096 | 40,441,112 | 211,770 |
